# KOS® Implants: A Case of Full Mouth Rehabilitation in One Day

**DOI:** 10.7759/cureus.119335

**Published:** 2026-11-08

**Authors:** Ralph Zarazir, Maryline Bitar, Georges Aoun

**Affiliations:** 1 Oral Surgery, Military Hospital, Beirut, LBN; 2 Oral Pathology, Saint Joseph University, Beirut, LBN; 3 Oral Medicine and Maxillofacial Radiology, Lebanese University, Beirut, LBN

**Keywords:** compressive, implant, kos, mouth rehabilitation, single piece

## Abstract

Full-mouth rehabilitation in edentulous patients remains a clinical challenge, particularly when bone resorption and prosthetic instability are present. This report discusses the case of a 56-year-old female patient with long-term edentulism and compromised removable prostheses who underwent extraction of the remaining anterior lower teeth and immediate placement of 22 single-piece compressive KOS® (Dr. Ihde Dental AG, Gommiswald, CHE) and basal cortical screw (BCS®; Dr. Ihde Dental AG) implants using a flapless approach. Within the same day, provisional polymethyl methacrylate bridges were delivered, restoring occlusion, aesthetics, and facial support. The patient reported superior comfort and stability compared to her previous removable prostheses. Immediate loading with compressive and basal implants provided predictable stability and rapid functional and aesthetic improvement, demonstrating the efficiency of modern implantology in complex edentulous cases.

## Introduction

The KOS® (Dr. Ihde Dental AG, Gommiswald, CHE) single-piece compressive implants are designed to achieve high primary stability through a unique design, drilling, and insertion protocol [1]. Unlike conventional implants that require extensive osteotomy preparation, KOS® implants rely on a minimally invasive approach with reduced drilling and flap elevation, if any. This allows the implant to compress the surrounding bone, enhancing mechanical retention and enabling immediate loading. The flapless technique further reduces surgical trauma, shortens operative time, and promotes rapid healing, making this system both efficient and predictable [1,2]. In this report, we present a case of a 56-year-old female patient with long-term edentulism and compromised removable prostheses who underwent extraction of the remaining teeth and immediate placement of KOS® and basal cortical screw (BCS®; Dr. Ihde Dental AG) implants using a flapless approach.

## Case presentation

A 56-year-old female patient, in good general health, presented with a full edentulous case, except for the lower anterior block, for more than 28 years (Figure 1, panels a and b). During this period, she compensated with a full upper denture and a partial lower denture (Figure 1, panel c).

**Figure 1:**
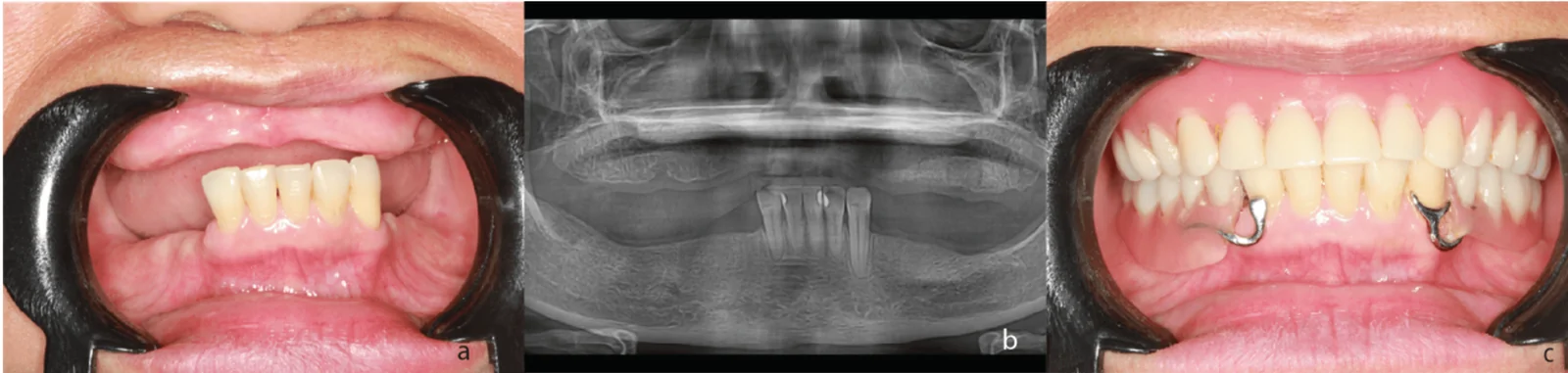
Intraoral view and X-ray a: Intraoral view of the remaining teeth; b: Preoperative panoramic X-ray; c: The existing removable prosthetics

While the removable prosthesis provided basic function, it was associated with instability, progressive alveolar bone resorption, and esthetic limitations. The patient expressed dissatisfaction with her smile and facial support (Figure 2, panel a) and sought a fixed solution that would restore both function and esthetics. The vertical dimension was recorded before the extractions to use the existing teeth as a reference (Figure 2, panel b).

**Figure 2:**
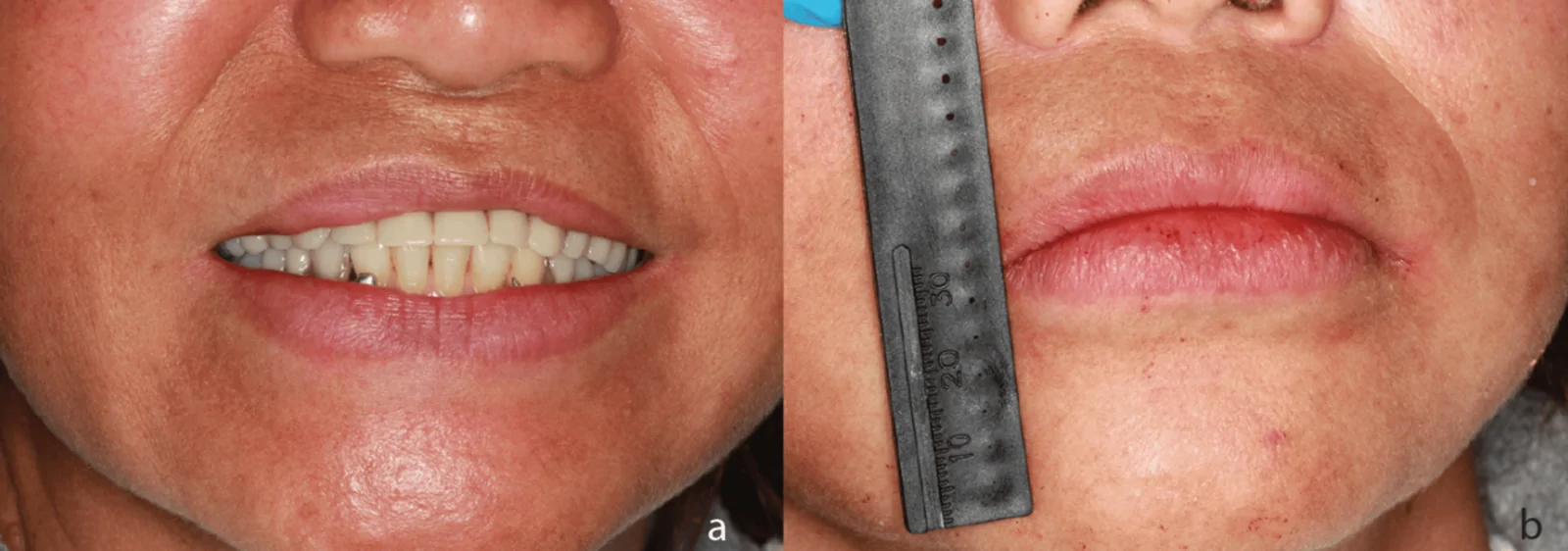
Negative smile line and compromised vertical dimension (a), and vertical dimension before the extractions (b)

After using local anesthetics, the lower antero-inferior teeth were extracted due to mobility, pain, and implant placement strategic reasons. A flapless surgical approach was adopted to promote faster healing and a predictable gingival level for the final prosthetics. Ten compressive implants were placed in the upper jaw, complemented by one basal implant (anchorage to the nasal floor) for additional stability (Figure 3). The space between implant positions 11 and 24 could not accommodate any additional compressive implant due to an ultra-thin ridge, so one additional BCS® implant made the pontic area shorter and thus enhanced the mechanics. Nine compressive and two basal implants were inserted in the lower jaw (Figure 3).

**Figure 3:**
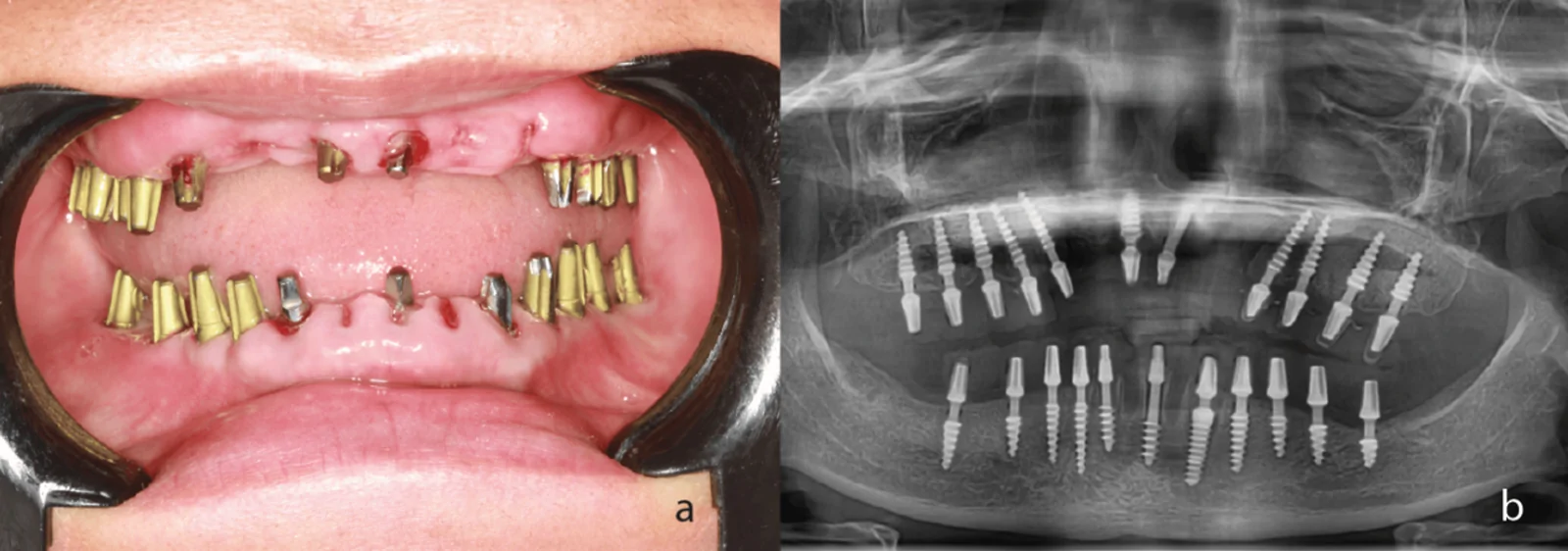
Panel 'a' shows the placement of 22 single-piece implants with extraction and immediate implantation in the anterior lower jaw. Panel 'b' features the panoramic radiograph after the placement of the implants. Longest implant: Tooth 21 (BCS, 20 mm length); Shortest implant: Tooth 37 (KOS Root, 6 mm length); Largest diameter: Tooth 33 (KOS Micro, 4.1 mm); Most common type: KOS Root (dominates across arches); BCS implants: Used mainly for anterior teeth (21, 31, 42) with longer lengths

The drilling protocol for KOS implants involves mostly minimal osteotomy preparation, typically limited to pilot drilling as a guide for implant placement. During insertion, the implant body advances into the bone, compressing trabecular structures and achieving high primary stability. This mechanical engagement is the cornerstone of the immediate loading concept, as it allows the prosthetic part to be supported without any waiting or healing period. Implant bending was performed on eight of the 22 implants whenever necessary to adjust the parallelism for prosthetic insertion reasons, mainly in the upper canine/premolar region and other sites as well. Different types, diameters, and lengths of implants have been used (Table 1).

**Table 1:** Types, diameters, and lengths of the implants used Longest implant: Tooth 21 (BCS, 20 mm length); Shortest implant: Tooth 37 (KOS Root, 6 mm length); Largest diameter: Tooth 33 (KOS Micro, 4.1 mm); Most common type: KOS Root (dominates across arches); BCS implants: Used mainly for anterior teeth (21, 31, 42) with longer lengths

Tooth #	Implant type	Diameter (mm)	Length (mm)
11	KOS Root	4.0	12
13	KOS Root	3.5	14
14	KOS Root	3.5	14
15	KOS Root	4.0	12
16	KOS Root	4.0	12
17	KOS Root	4.0	10
21	BCS	3.5	20
24	KOS Root	3.5	14
25	KOS Root	3.5	12
26	KOS Root	4.0	12
27	KOS Root	4.0	10
31	BCS	3.5	17
33	KOS Micro	4.1	15
34	KOS Root	3.5	12
35	KOS Root	3.5	8
36	KOS Root	4.0	8
37	KOS Root	4.0	6
42	BCS	3.5	14
43	KOS Root	3.5	14
44	KOS Root	3.5	12
45	KOS Root	4.0	8
46	KOS Root	4.0	10

Immediately after the surgery, impressions were taken using heavy and light body materials to capture precise details after splinting the impression caps using flowable composite with perfect fitting to the abutment head (Figure 4, panel a). Right after, the plastic double-sided analogues were inserted into the impression caps, depending on the small head or large head size of the implant (Figure 4, panel b). The wax bite was registered on the newly placed implant heads, elevating the vertical dimension by 1.5 mm, defining the midline, the canine line, and the shade of the upcoming teeth (Figure 4, panel c).

**Figure 4:**
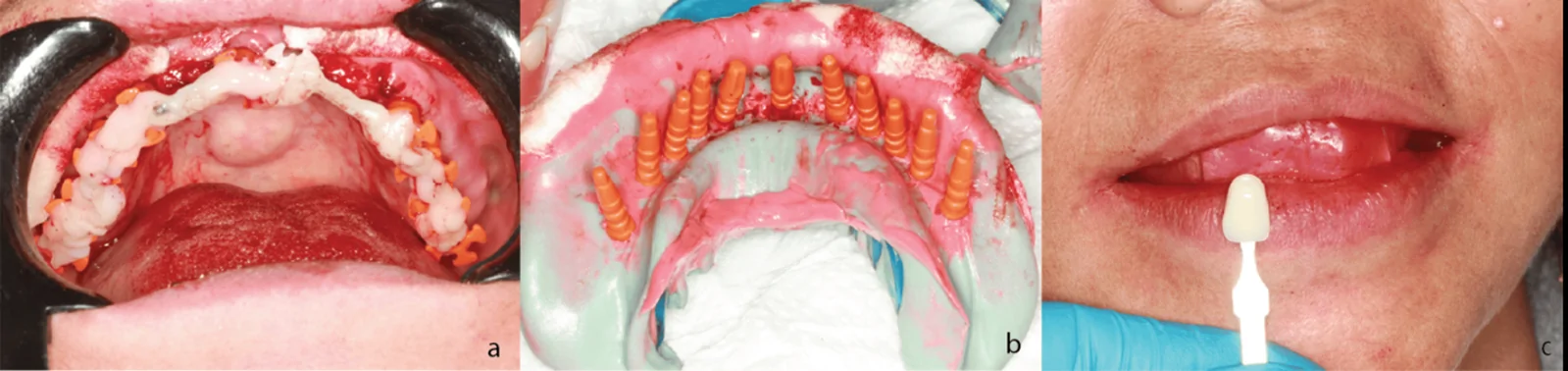
Panel 'a' shows the impression caps splinting using flowable composite with perfect fitting to the abutment head; panel 'b' showcases the insertion of the double-sided analogues; and panel 'c' features the registration of the bite on the implant heads, defining the midline and shade of the upcoming teeth.

The models were scanned (Figure 5, panel a), and the polymethyl methacrylate (PMMA) bridges were milled according to a precise 3D simulation adapted to the patient’s facial shape & esthetic demands. Prosthetics adapted to the gingival margins were fabricated (Figure 5, panel b).

**Figure 5:**
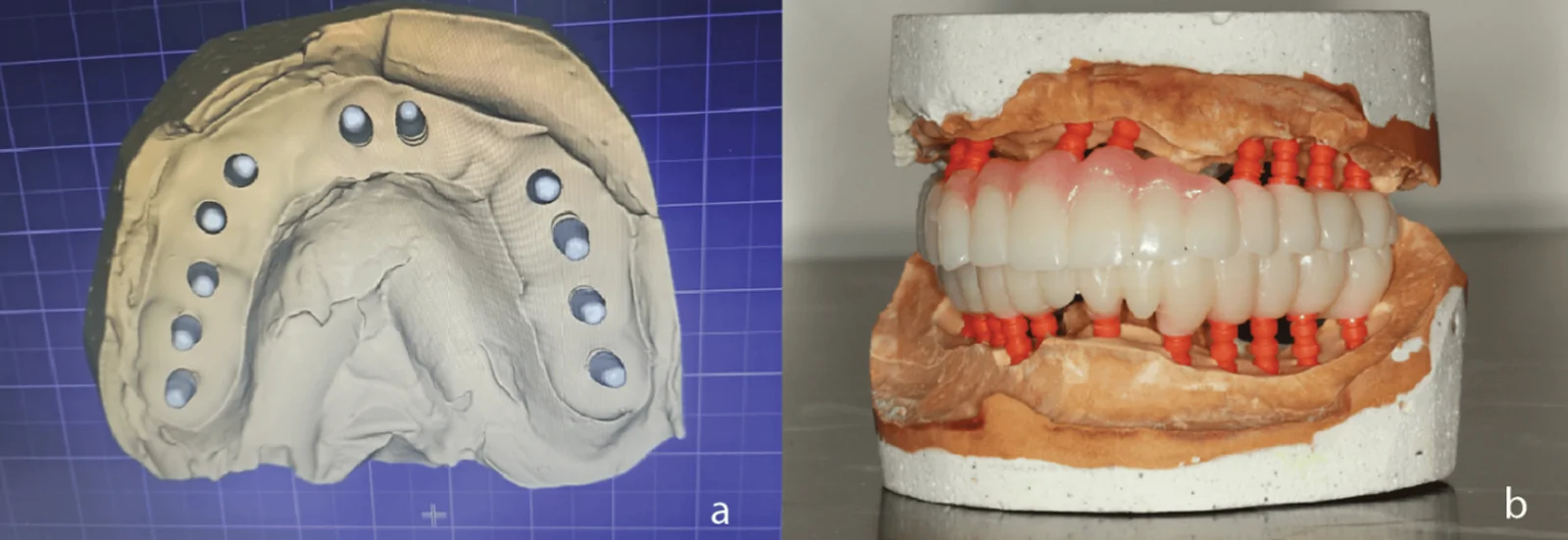
Occlusal view of the 3D scan of the upper jaw (a) and prosthetics adapted to the gingival margins (b)

Final zirconia or porcelain-fused-to-metal bridges could have been placed, but it was decided to keep a long-term temporary solution due to the extractions in the anterior lower jaw and then make a final decision after a period of six months to observe the crestal bone resorption post-extraction. On the same day, the patient’s teeth were cemented, designed to restore occlusion, esthetics, and facial harmony. Extraoral and intraoral photos were taken showing the hygienic margins of the new crowns. Functionally, the patient reported a remarkable superior comfort and stability compared to her previous removable solution. Her final smiling photography shows her physical and psychological improvement (Figure 6).

**Figure 6:**
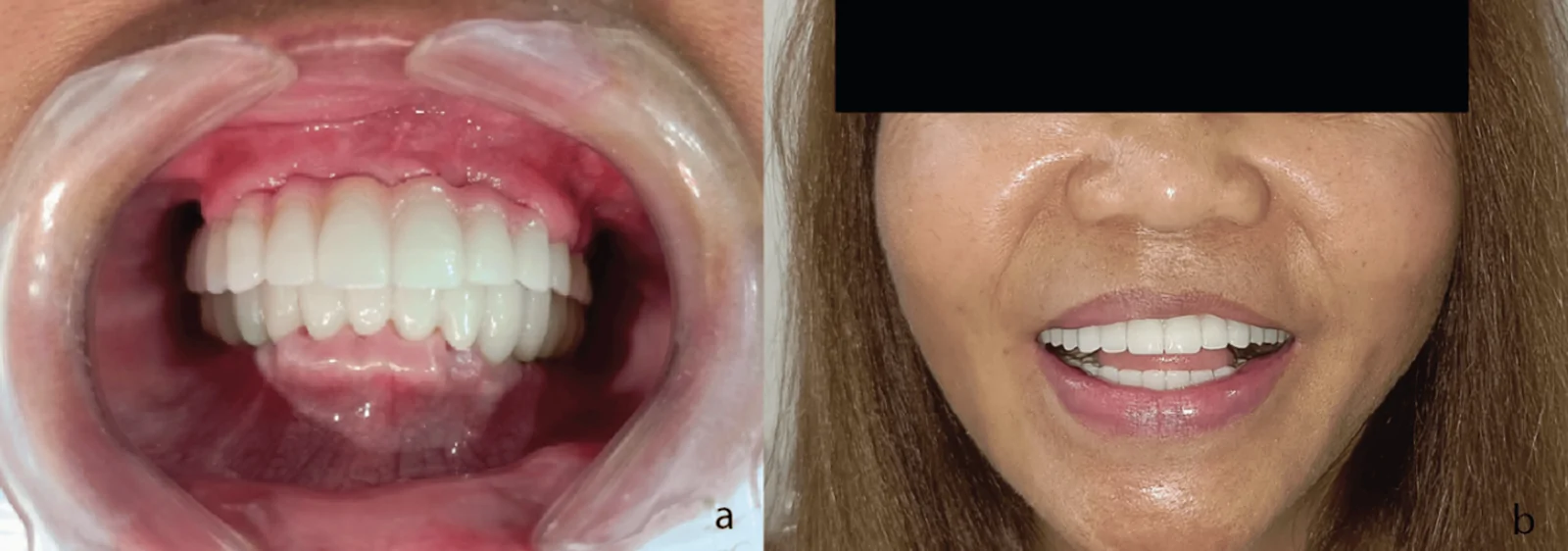
Intraoral photo showing the hygienic margins of the new crowns (a) and the final ‘smiling’ result (b)

The patient experienced a dramatic improvement in her incisal line and smile curve (Figure 7). The fixed prosthesis provided enhanced facial support, resulting in a natural facelift effect. The patient went from having a sad smile to a positive smile curvature (Figure 7). The immediate loading protocol eliminated the prolonged waiting period without stable dentition, traditionally associated with implant therapy, allowing her to resume normal activities right after the procedure.

**Figure 7:**
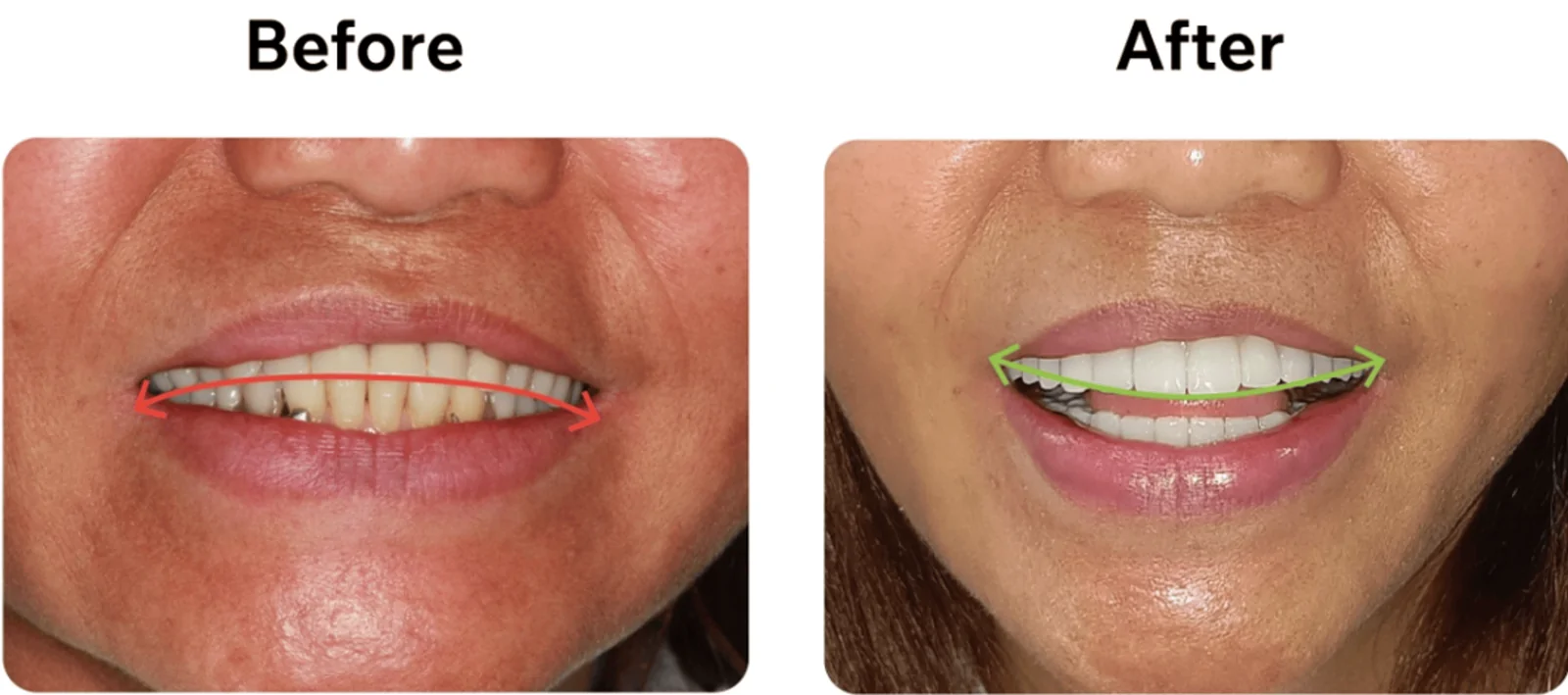
Before and after with the red and green curved lines showing smile line enhancement

## Discussion

This case highlights the biological and prosthetic advantages of single-piece compressive implants compared to conventional two-piece systems. In traditional implantology, repeated manipulation of abutments and transfer copings can disrupt gingival fiber healing, compromising the peri-implant soft tissue seal. The one-piece design of KOS® implants minimizes working steps, preserves gingival integrity, and reduces chairside manipulation, thereby enhancing predictability and patient comfort [1]. More than half of all installed two-stage implants face the problem of peri-implantitis, even after two, three, or four years of treatment completion [2].

A critical advantage lies in the absence of a micro-gap between implant and abutment. Two-piece implants inherently present a junction that harbors bacterial adhesion, which has been linked to peri-implantitis and marginal bone loss. Consensus reports confirm that rough implant surfaces are particularly prone to bacterial colonization, increasing the risk of peri-implant disease [3,4]. The monolithic structure and laser surface of KOS® implants eliminate this interface, offering a smooth, biocompatible surface that supports long-term peri-implant health [5,6].

The flapless surgical approach used in this case preserved peri‑implant soft tissue architecture and minimized crestal bone resorption. Studies confirm that flapless immediate implant placement maintains vascular supply to the periosteum, reducing surgical trauma and promoting stable gingival margins [7]. This biological preservation is critical for long‑term esthetics and function.

Immediate loading protocols have demonstrated high success rates in full‑arch rehabilitations. Ihde and Sipic reported survival rates exceeding 95% for basal and compressive implants under immediate loading [8]. Agliardi et al. documented predictable outcomes in edentulous jaws rehabilitated with fixed prostheses supported by implants [9]. Recent systematic reviews confirm that immediate loading protocols can achieve survival rates comparable to delayed approaches, with reduced marginal bone loss in fresh sockets [10]. Clinical case series further demonstrate that immediate placement and provisionalization in the anterior maxilla yield stable soft‑tissue contours and high esthetic scores when prosthetically guided [11].

The use of provisional PMMA bridges for six months allowed monitoring of ridge resorption following extractions. Literature indicates that alveolar bone resorption is most pronounced within the first three to six months post-extraction, with rates up to 40% in vertical height [12]. By delaying definitive zirconia crowns, prosthetic margins were maintained in a hygienic position, ensuring long-term stability and esthetics.

Basal implants provided supplementary anchorage in thin ridges and the nasal floor, reinforcing mechanical reliability. Evidence supports their role in eliminating the need for grafting and enabling immediate functional loading even in severely resorbed jaws [8]. Overall, this approach demonstrates how single‑piece immediate loading implants, combined with flapless surgery and provisionalization, can overcome the limitations of removable prostheses, reduce biological complications associated with micro-gaps and rough surfaces, and deliver predictable, efficient, and durable outcomes in full‑mouth rehabilitation.

## Conclusions

This case demonstrates the effectiveness of immediate loading with compressive KOS® and few basal BCS® implants in rehabilitating an edentulous patient. The disadvantages of removable prostheses, including instability, bone resorption, and social discomfort, were successfully overcome. The compressive drilling protocol of KOS® implants provided excellent primary stability, enabling immediate loading and rapid esthetic transformation. The flapless technique simplified the procedure, making it straightforward and reliable for both the dentist and the patient. This approach highlights how modern implantology can deliver predictable, efficient, and life-changing outcomes.
